# Water Intake, Body Water Regulation and Health

**DOI:** 10.3390/nu12030702

**Published:** 2020-03-06

**Authors:** Evan C. Johnson, William M. Adams

**Affiliations:** 1 Human Integrated Physiology Laboratory, Division of Kinesiology and Health, University of Wyoming, Laramie, WY 82071, USA; 2 Hydration, Environment and Thermal Stress Lab, Department of Kinesiology, University of North Carolina at Greensboro, Greensboro, NC 27412, USA; wmadams@uncg.edu

The biological feedback provided by human water intake upon our physiology is grossly under-investigated. The delicate regulation of intake and imperceptible changes to physiological processes makes it easy for the casual observer to overlook the acute and chronic impacts of water consumption on human health and performance. Given this gap, we aim to bring a special edition of Nutrients to highlight some of the growing areas of interest that fall under the broad umbrella of “water intake, body water regulation and health”.

As with any research topic, investigators must begin with, and be able to constantly update, their understanding of the appropriate measurement of their target phenomenon. Three of the manuscripts within this Special Issue will help the researchers of tomorrow to do just that. Drs. Muñoz and Wininger provide us with “food for thought” when considering the utilization of the National Health and Nutrition Examination Survey (NHANES) for hydration-related investigations [1]. Additionally, Dr. Basov and colleagues review the influence of deuterium-depleted water on isotope regulation, an important topic for those looking to apply D_2_O application for the measurement of water intake and/or turnover [2]. Relatedly, Dr. González-Arellanes and co-authors present evidence of how chronic high-volume water consumption can affect body composition measurement via D_2_O dilution while also introducing the influence that age, sex, and ethnicity may play in being able to accurately assess total body water [3]. 

Although still under-investigated, the influence of water intake on dimensions of health has been increasing within recent literature. Rightly so, a further understanding of health behaviors related to something as fundamental as water intake can have a substantial impact on public health. First, Drs. Watso and Farquhar provide a comprehensive review discussing current evidence and physiological mechanisms that tie water intake to cardiovascular function [4]. A separate study presented by Drs. Sollanek, Kenefick, and Cheuvront reviews the osmolality standards of several commercially available rehydration solutions [5], which has relevance to treatment standards within countries in need of rehydration plans for combatting diseases such as malaria. Tangential to water intake, is the influence that body water has upon the human body’s ability to thermoregulate. Papers by Dr. Schlader and colleagues [6] and Dr. Smith [7] address this aspect of water homeostasis through a mechanistic review of how physical labor in the heat presents risk for renal injury, and a novel focus regarding pediatric thermoregulation in the face of increasing ambient temperature due to climate change, respectively. Lastly, among our health focused papers, is a wonderful review by Dr. Hew-Butler which examines the other side of the water intake coin; what happens to the body when too much water is consumed [8]? The career achievements of all of the above authors make these manuscripts not to be missed for those interested in the influence of water intake on health.

In conclusion, the co-editors of this Special Issue, together with our collaborators, introduce two papers on hydration biomarkers; a topic that is continuously evolving within scientific literature. The manuscript by Drs. Armstrong and Johnson provides background on the evidence behind the current water intake guidelines and introduces a novel biomarker, copeptin, along with providing the history of how this biomarker came to be established in the water intake literature [9]. Drs. Adams, Vandermark, Belval, and Casa present our final manuscript on how the perception of thirst can be properly used to evaluate hydration status within exercise investigations [10]. To read this paper alongside Dr. Hew-Butlers will provide fantastic context for early-career scientists to emeritus scientists.

We thank the readers for seeking out this Special Issue. We are honored to be able to collect the works from a diversified group of leaders within the field of human physiology. The data, thoughts, and ideas presented in this Special Issue are a sign of wonderful times ahead in our field!
